# Can the narrow red bands of dragonflies be used to perceive wing interference patterns?

**DOI:** 10.1002/ece3.4054

**Published:** 2018-05-08

**Authors:** Mikkel Brydegaard, Samuel Jansson, Marcus Schulz, Anna Runemark

**Affiliations:** ^1^ Department of Physics Lund University Lund Sweden; ^2^ Norsk Elektro Optikk AS Skedsmokorset Norway; ^3^ Department of Biology Lund University Lund Sweden; ^4^ Agilent Technologies GmbH Waldbronn Germany; ^5^ Department of Biosciences University of Oslo Oslo Norway

**Keywords:** mate choice, Odonata, private channels, sexual signaling, visual ecology, wing interference patterns

## Abstract

Despite numerous studies of selection on position and number of spectral vision bands, explanations to the function of narrow spectral bands are lacking. We investigate dragonflies (Odonata), which have the narrowest spectral bands reported, in order to investigate what features these narrow spectral bands may be used to perceive. We address whether it is likely that narrow red bands can be used to identify conspecifics by the optical signature from wing interference patterns (WIPs). We investigate the optical signatures of Odonata wings using hyperspectral imaging, laser profiling, ellipsometry, polarimetric modulation spectroscopy, and laser radar experiments. Based on results, we estimate the prospects for Odonata perception of WIPs to identify conspecifics in the spectral, spatial, intensity, polarization, angular, and temporal domains. We find six lines of evidence consistent with an ability to perceive WIPs. First, the wing membrane thickness of the studied Odonata is 2.3 μm, coinciding with the maximal thickness perceivable by the reported bandwidth. Second, flat wings imply that WIPs persist from whole wings, which can be seen at a distance. Third, WIPs constitute a major brightness in the visual environment only second after the solar disk. Fourth, WIPs exhibit high degree of polarization and polarization vision coincides with frontal narrow red bands in Odonata. Fifth, the angular light incidence on the Odonata composite eye provides all prerequisites for direct assessment of the refractive index which is associated with age. Sixth, WIPs from conspecifics in flight make a significant contribution even to the fundamental wingbeat frequency within the flicker fusion bandwidth of Odonata vision. We conclude that it is likely that WIPs can be perceived by the narrow red bands found in some Odonata species and propose future behavioral and electrophysiological tests of this hypothesis.

## 1 INTRODUCTION AND BACKGROUND

**Figure 1:**
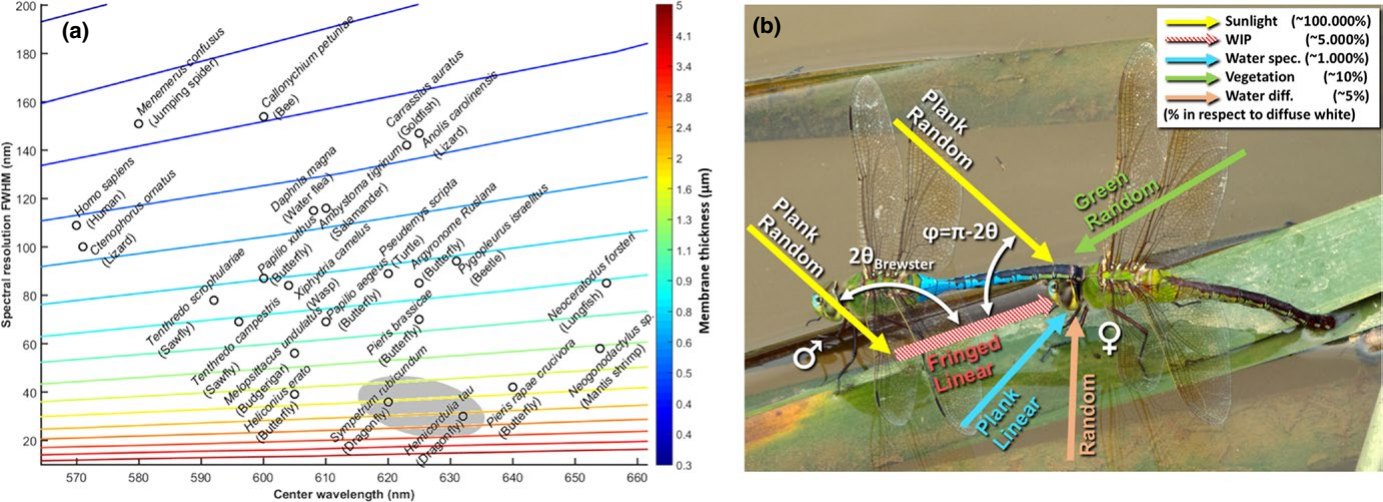
(a) Cross species scatterplot of photoreceptor sensitivity center wavelength and spectral width with focus on species with particularly long‐waved bands. Only the longest bands are reported for each species. The colored lines indicate fringe from membrane thicknesses at Brewster angle given by the color bar. We note that Odonata (here *Sympetrum rudicundulum* (Meinertzhagen et al., 1983) and *Hemicordulia tau* (Yang & Osorio, 1991)) are expected to be able to resolve the thickest membranes based on the properties of their visual systems. For detailed list of references, see list in complimentary material. (b) Damselfly tandem, where the male (left) has clasped the female's prothorax prior to formation of a copula. The arrows indicate light propagation of various visual contributions, whereas the labels indicate spectral and polarization properties. Coarse intensity estimates are given in the legend. These estimates give an idea of the significance of wing interference patterns in relation to the background. The percentage numbers are relative to a diffuse Lambertian white reference

Animal vision differs from ours with respect to the numbers and central wavelengths of the spectral bands. Variation in ultraviolet coverage and the number of spectral bands are well studied (Cronin, Johnsen, Marshall, & Warrant, 2014). In contrast, we know little about the variation in spectral bandwidth. Generally, UV bands are the narrowest, and red bands are some 50% broader (Maximov, 1988; Peitsch et al., 1992). Nevertheless, narrow red bands of Odonata reported are 32 nm broad, four times narrower than expected from Maximov and Peitsch's reportings (see values in Figure 1a; Maximov, 1988; Meinertzhagen, Menzel, & Kahle, 1983; Peitsch et al., 1992; Yang & Osorio, 1991). Most bands are broad and capture photons with a wide range of energies. This is sufficient for contrasting most spectral features in the environment (Campbell, 2011; Cronin et al., 2014). Nevertheless, while certain dragonflies have the narrowest bands reported from the animal kingdom, there is no explanation to the benefits of narrow bands. We investigate the possibility that they use these bands to perceive wings of other dragonflies—the only natural objects reflecting such narrow band light.

There are few reasons to expect narrow spectral vision bands. Vegetation and soil have dull spectra (Campbell, 2011) for which the main features are easily resolved by ordinary polychromatic vision (Cronin et al., 2014). Solar and terrestrial atmospheric gases produce sharp Fraunhofer lines, but these are either bulk constituents with little variance on the relevant spatial scale of insects or trace gases so dilute that optical sensing is by far inferior to other receptors (Blahó et al., 2013; Bowen, 1991; Maekawa et al., 2011; Majeed, Hill, & Ignell, 2014). Also, rare earths can exhibit narrow absorption features (Svanberg, 2004), but has little ecological relevance. Yet, it has been demonstrated that several species have narrow bands down to 60 nm full‐width half‐maximum (FWHM), using long‐pass absorption filtering at the expense of reduced sensitivity (Hart & Vorobyev, 2005; Warrant & Locket, 2004). Even the polychromatic masters, stomatopods have bands down to 60 nm FWHM, see, for example, *Neogonodactylus* sp. in Figure 1a. Odonata, however, have widths down to only 30 nm FWHM. Thin films of insect wings are the parts of the visual surroundings of Odonata which can produce spectral features of a width in the order of 30 nm. In comparison, no features of this sharpness are encountered by vegetation (Blackburn, 2006; Thenkabail & Lyon, 2016) and absorption of organic pigments or chromophores do not exhibit such sharp spectral features (Hill & McGraw, 2006a; Popp, Tuchin, Chiou, & Heinemann, 2011). Structural interference colors are common in insects and Odonata, and they arise from dominant spatial frequencies of refractive index of submicron organized structures or organelles (omochrome granules) and exhibit spectral features of some 150 nm width (Nixon, Orr, & Vukusic, 2013, 2015; Shawkey et al., 2009). Spectral features relate inversely to spatial features (inherent in the Fourier transform), and sharp spectral features of 30 nm width require interference between spatial features of a couple of micrometers. Wing membranes produce dominant spatial frequencies in one dimension along the surface normal (Stavenga, 2014). Thick insect wing membranes are therefore likely to produce the sharpest spectral details in the visual environment of animals. For comparison, the narrowest spectral details presented in the Visual Ecology book (Cronin et al., 2014) is 70 nm broad. In this reasoning, we exclude sharp spectral content of biological tissue that can be enhanced by technological equipment, for example, optical coherence tomography (Backman et al., 1999; Walther et al., 2011). Through investigating the information that can be perceived with bands of different widths, we can start understanding the biological implications of, and reasons for, bandwidth variation.

Odonata, the order of dragonflies and damselflies, are aerial predators hunting by sight (Combes, Rundle, Iwasaki, & Crall, 2012) and are often brightly colored in the adult stage (Corbet, 1999). Coloration is important for partner search image (Fincke, 1997; Le Rouzic, Hansen, Gosden, & Svensson, 2015; Sánchez‐Guillén, Córdoba‐Aguilar, Cordero‐Rivera, & Wellenreuther, 2014), for mate choice (Svensson, Eroukhmanoff, Karlsson, Runemark, & Brodin, 2010; Svensson, Karlsson, Friberg, & Eroukhmanoff, 2007), and for identifying competitors (Tynkkynen, Rantala, & Suhonen, 2004). There are several stages during which Odonata observe and identify potential partners as well as rivals where they possibly could evaluate the wing surface properties by exploiting specular reflections. Males of some Odonata species are territorial (Tynkkynen, Kotiaho, Luojumäki, & Suhonen, 2006) and could potentially identify or intimidate competitors based on specular reflections. Males first identify females at a distance and court or chase them (Gosden & Svensson, 2009). Here, specular reflections from entire wings could aid males to identify conspecific females and females to avoid heterospecific males.

Mate choice is mutual in many Odonata species (Fincke, 1997; Svensson, Runemark, Verzijden, & Wellenreuther, 2014), and mating typically requires cooperation from the females (Fincke, 1997) in this taxon. After a tandem is formed (Oppenheimer & Waage, 1987; Runemark, Wellenreuther, Jayaweera, Svanberg, & Brydegaard, 2012; Svensson, Nordén, Waller, & Runemark, 2016), the male encourages the female to bend up her abdomen to form a copula (Futahashi, 2016), and at this stage, the female can resist or accept mating (Fincke, 1997). Female mate choice based on male wing pigmentation is well established in, for example, calopterygid damselflies (Córdoba‐Aguilar, Salamanca‐Ocaña, & Lopezaraiza, 2003; Siva‐Jothy, 1999; Svensson et al., 2010, 2014). Potentially, species‐specific WIPs could also be used as a species recognition cue at this stage. When the couple are in tandem (see Figure 1b), the female is able to inspect the male's wings from a close range, as they are perpendicular with respect to his abdomen. The wings are flat membranes, and the surface of the wings is horizontally positioned in resting dragonflies (Corbet, 1999). Odonata typically mate in full daylight, under clear sky conditions, and hence are exposed to 90% direct broadband sunlight. The sunlight is collimated to the extent of the aperture of the solar disk (the spread of incident angles is ±¼°). If the tandem is facing the sun, the female will experience a strong specular reflection from the male's wing membranes. In fact, the magnitude of the specular reflection can be ten times higher than the diffuse reflectance from a perfectly white Lambertian reflector (Schultz & Fincke, 2009). Against a vegetation background that typically has a diffuse reflectance below ten percent, these specular reflections are hence a major contribution to the optical signature that the female perceives.

Here, we test whether the spectral properties of Odonata wings are such that it is likely that the narrow red band could be used to retrieve information from wings of conspecifics using a male *Cordulegaster boltonii* as a study species. In order for a specular reflex or WIPs signal to be perceived by a conspecific, the emitted optical signature must be resolved by Odonata vision in all optical domains. We address whether the optical properties of Odonata wings in the (1) spectral, (2) spatial, (3) intensity, (4) polarization, and (5) temporal domains are consistent with the ability to resolve them for *Sympetrum rudicundulum* (Meinertzhagen et al., 1983) and *Hemicordulia tau* (Yang & Osorio, 1991) which have narrow red bands. We use several methods to address the prospect of Odonata perceiving Odonata wing interference patterns (WIPs) in the above‐mentioned domains, including two theoretical and four experimental techniques. In the methods, we revise how these were used to investigate the prospects for visual signaling of Odonata wing interference patterns (WIPs). In the Results section, we present the optical properties of Odonata wings for each domain and compare the findings to what is known about Odonata vision.

## 2 METHODS AND TECHNIQUES

This study is based on a *Literature Review*,* Thin Film Calculations*,* Hyperspectral Imaging*,* Laser Profiling*,* Ellipsometry,* and In Situ *modulation spectroscopy*. We here revise how these tools were used for our assessment in the (1) spectral, (2) spatial, (3) intensity, (4) polarization, and (5) temporal domains. We elaborate on the technical details below this overview section.

1. *Spectral domain*: To evaluate the potential of Odonata spectral perception of WIPs, we compare spectral bandwidths from a *Literature Review* to the thickest membrane that can be perceived with the bandwidths in Odonata using *Thin Film Calculations*. We then apply *Hyper Spectral Imaging* to measure the spectral reflectance across Odonata wings. To evaluate whether whole wings produce a perceivable spectral signature when integrated over the surface we use *Ellipsometry*.
2. *Spatial domain*: To estimate to what extent WIPs are resolved spatially, we compare spatial resolution of Odonata vision from the literature to the spatial variance of thicknesses using *Hyperspectral Imaging*. To evaluate whether the WIP signal from whole wings could be mediated over distances, we assess the wing flatness using *Laser Profiling*. We eventually confirm that whole wings can produce WIPs using *Ellipsometry*.
3. *Intensity domain*: In order to assess the WIP signal intensity strength and relevance in natural conditions, we evaluate the quantitative diffuse reflectance values from *Hyperspectral Imaging* and compare them to common values in the visual environments. The reflectance estimates are later confirmed from *Ellipsometry*.
4. *Polarization domain*: To compare polarimetric vision of Odonata to the polarization properties of Odonata WIPs, we compared polarization properties from *Thin Film Calculations* and *Ellipsometry* to Odonata polarization vision described in literature. The assessment is later confirmed by polarimetric In Situ *Modulation Spectroscopy*.
5. *Temporal domain*: Finally, we estimate whether the flashing WIPs signals can be perceived in the temporal domain. To do this, we present remotely sensed optical signals from free‐flying insects and Odonata using instrumentation that we refer to in this article as In Situ *Modulation Spectroscopy*.

### 2.1 Literature review

Data from several animal vision review articles were compiled (Briscoe & Chittka, 2001; Bybee, Johnson, Gering, Whiting, & Crandall, 2012; Futahashi et al., 2015; Kelber, Vorobyev, & Osorio, 2003), and spectral bands of particularly long central wavelength were identified from meta studies and original papers (Meinertzhagen et al., 1983; Yang & Osorio, 1991). The FWHM was extracted from the graphics displaying long‐wave band response recorded through electrophysiography, and special attention was given to the linearity of the sensitivity scale. The width was measured by caliper and rescaled to wavelength axis. In the case of the spectral bands of *Hemicordulia tau* (Yang & Osorio, 1991), the bands reported in a graph were stored in a digital image, the curve points were digitized by a custom Matlab^®^ script, and multi‐Gaussian fit was applied to each band, providing an analytical function for each band. There are two reports of narrow far red bands, namely *Sympetrum rudicundulum* (Meinertzhagen et al., 1983) and *H. tau* (Yang & Osorio, 1991). Similar bands have been encountered in other species (personal communication with Almut Kelber, Lund Vision Group). There can be a number of reasons why far red narrow bands are not reported in all studies, including spectral range of investigation and step size of spectral sampling, and many studies focus on one particular aspect or one particular region of the eye. The fact that a vision study on Odonata does not report a far red narrow band does not therefore necessarily exclude the presence of one.

### 2.2 Thin film calculations

Optical thin film was described in the seventeenth century by early physicists, for example, Robert Hooke and Isaac Newton (Kinoshita & Yoshioka, 2005). Further advancing the understanding of wave and polarization optics, Augustin‐Jean Fresnel and David Brewster from the eighteenth century provided quantitative equations for reflected intensity from surfaces as a function of incidence angle and polarization. Together, thin film interference and the Fresnel equations provide a complete description of reflectance, R, from thin films given in relation to incidence angle, θ, polarization state (S or P), refractive index, *n*, wavelength, λ, and membrane thickness, d. These numerical and analytical calculations of wing interference patterns are found in literature, for example, Yin et al. (2006). In the reflected spectrum, such interference appears as an oscillation and we refer to each reflectance peak as a spectral fringe. The wavelength‐dependent refractive index for chitin (Leertouwer, Wilts, & Stavenga, 2011) of *n*_(λ)_ = 1.517 + 8,800 nm^2^/λ^2^ was employed. The refractive index only varies 2% over the visible range, and the Brewster angle, θ_Br_ = tan^−1^(*n*), only changes less than 1° across the spectrum, though. A weak melanization absorption was included in the modeled spectra (Jacques, 2013). In our case, we found this absorption to be μ_abs_ = C λ^−3.48^, where C = 4 × 10^8^ μm^−1^. The effect of melanization somewhat resembles that of gradient refractive index (Stavenga, 2014), which we have not included in this report.

The width of spectral fringes (FWHM) was estimated numerically in the far red region where the narrow red band is encountered. Membrane thickness from specular hyperspectral pixels was determined by projecting data on a function space of spectral fringes (saturated hyperspectral bands are excluded) with a constant periodicity in the light frequency domain (reciprocal wavelength scale). This is equivalent to a Fourier transform in the light frequency domain. Thickness was identified by the most predominant light frequency.

### 2.3 Hyperspectral imaging

The hyperspectral imaging was performed using Hyspex, hyperspectral bush broom cameras from Norsk Elektro Optikk AS. Both a VIS‐NIR instrument (Si‐CCD) covering 400–1,000 nm and a SWIR instrument (HgCdTe) covering 900–2,500 nm were used. We only present data from the visible and near infrared. The near‐infrared parts are included as these spectral features displace into the visible region when the angle of incidence increases. The images are white calibrated using a gray reference (Spectralon^®^) and are flat field calibrated using the same material. This material has spectrally flat reflectance of 50% and reflects diffusely close to Lambertian (~cos(θ)). Because of the normalization to a diffuse Lambertian target, the resulting reflectance from specular pixels may greatly exceed 1 (we encountered specular reflections up to 4,000% compared to Lambertian white). Reflectance of diffuse objects (including vegetation) is in the order of ~10%.

The imaged male *Cordulegaster boltonii* is a museum specimen mounted with needles on carbon filter foam board with minimal reflectance. The chitin polymers in the wings of Odonata are known to harden and alter appearance and glossiness the first few days after emerging. Hereafter, we do not expect any major changes to the WIPs. In one study, WIPs were shown to be preserved over 100 years (Shevtsova, Hansson, Janzen, & Kjaerandsen, 2011). The cameras were mounted on a linear translation stage and were scanning along the insect boards with the optical axes perpendicular to the boards. The swat width is 10 cm and resolved by 1,600 pixels for the VIS‐NIR instrument.

The illumination is provided by a 100‐W, 10‐cm tungsten–halogen rod filament with a parabolic aluminum reflector (~8° light cone angle). The light impinges at 45°, and thus, the condition for specular reflection is met when target surface normal is θ = 22.5° with regard to the optical axis of the camera. The angular illumination field is verified by imaging Teflon, chrome, and silicon nitride spheres; for details, see Debevec et al. (2012). These measurements confirm that the angular illumination distribution is close to a point source at 45° and deviates minimally over the swat width.

### 2.4 Ellipsometry

One forewing of the male *C. boltonii* was positioned in an automated hyperspectral polarimetric goniometric ellipsometer (model Cary 7000 UMS from Agilent Technologies). This machine, resembling a spectrometer, is able to rotate the sample as well as the detector in relation to a monocromatized light source. Furthermore, the light source can be polarized in an arbitrary direction. Reflectance and transmittance were recorded in the spectral span λ = 250 nm to 2,500 nm. The sample was measured for a range of incidence angles of θ = 10° to 70°. Both S‐polarized and P‐polarized reflectance were recorded with both co‐ and depolarized detection. The incoherent diffuse reflectance was assessed through depolarization, employing cross‐polarized detection in the ellipsometer. For details, see Jacques (2016).

The entire data can be downloaded from the [Link], [Link], [Link], [Link], [Link]. Initial observations are the incoherent contribution from S‐polarized and P‐polarized light to the absolute reflectance scales linear with the S‐polarized coherent component. Diffuse contribution can be expressed roughly as *R*_diff_ = 6.6∙10^−5^ + 1.2∙10^−4^∙θ^4^. For comparison, the specular absolute reflectance reached 0.5% @ θ = 10° and 7% @ θ = 70°; therefore, optical signatures from *C. boltonii* and most other dragonflies are dominated by the specular contributions on which this report focuses. We also note that these ratios from absolute reflectance from ellipsometry are in correspondence with the diffuse reflectance ratios from diffuse and specular reflections in hyperspectral imaging.

We confirmed that the observed Brewster angle corresponds to that encountered for chitin, θ_Br_ = tan^−1^(n_chitin_) = 57°. The degree of linear polarization (DoLP) showed similar angular dependence in ellipsometry as from the Fresnel equations. However, the experimental values only reached 85% DoLP compared the theoretical value of 100% at Brewster angle. Whole‐wing WIP fringes were observed across the spectral span, with the shortest discernable fringes starting from blue light 460 nm.

The absolute reflectance increases dramatically when incidence angle exceeds the Brewster angle but the fringe modulation depth (peak‐valley contrast) is preserved for the various incidence angles. The fringe modulation depth is roughly the same for S‐ and P‐polarized reflectance, but the modulation increases with the wavelength, λ. Around λ = 2 μm modulation depth exceeds 60% but around 630 nm modulation depth is some 12%. There are several reasons why modulation depth is smaller for shorter wavelength and for the whole‐wing ellipsometry than for individual pixels in the hyperspectral imaging. The surface flatness in relation to wavelength increases for longer waves, also the sharp fringes in the short region also shift with small surface normal deviations from the wing normal; thus, the spectral signatures from all the wing segments may not interfere constructively when integrating over the wing.

As a final verification, the transmittance displayed the weak imprint of the displacing fringes for the various angles, *T* = 1−R. Transmittance also showed the characteristic chitin absorption dip remaining at 280 nm regardless of incidence.

### 2.5 In situ modulation spectroscopy

Our group has a previously remotely measured backscatter from airborne insects with kHz sample rates (Brydegaard, 2015; Brydegaard, Gebru, & Svanberg, 2014; Brydegaard, Merdasa, Gebru, Jayaweera, & Svanberg, 2016; Gebru, Brydegaard, Rohwer, & Neethling, 2016; Malmqvist, Jansson, Torok, & Brydegaard, 2016). To retrieve optical modulation spectra in the laboratory experiment, the light from a 808‐nm laser diode was collimated and transmitted 5 m across the room. A Si photodiode placed next to the laser source was aligned so that its field of view (FOV) overlapped the laser beam at an approximate distance of 2.6 m. Both the laser beam and the detector FOV were terminated with dark cavities built from neoprene to minimize background light. Fruit flies *Drosophila melanogaster* were released in the overlap volume, which constitutes the probe volume of the experiment. The backscattered light from released insects was recorded at 20 kHz, yielding detailed wingbeat waveforms of the scattering cross section from the entire organism. The specular reflections show up as spikes on the waveform in the time domain or as high harmonics in the frequency domain. We have observed these in flight specular reflections in a wide range of insects with glossy wings both in field and in laboratory, but have only recently been able to resolve them polarimetrically. The bandwidth of the detectors in the present setup is 3.4 kHz (−3 dB). Bandwidth is always a limiting factor in resolving specular reflections, but it is reasonable to assume that at least an equal number of harmonics from specular reflections would pertain to other species. We also recorded the highest in flight fundamental wingbeat frequency, 960 Hz for *Culex* mosquito males at 32°C, which would produce modulation frequencies of at least 25 kHz (Gebru et al., 2018).

We recorded the modulation spectrum from a dragonfly at 130 m distance using remote dark field spectroscopy. The method and site are described in Runemark et al. (2012), and the kHz modulation variety and calibration are described in Brydegaard et al. (2016). The particular observation presented is confirmed to a male *Anax imperator* by video (Salman, 2012). The remote dark field method currently does not allow polarimetric investigation, and diffuse contribution is estimated from the waveform.

Polarimetric entomological lidar was carried out over a rice pond in southern China (Zhu et al., 2017). Similar polarimetric lidar is described in Mei, Guan, Yang, and Kong (2017). The presented polarimetric observation is presumed to be an Odonata because of (1) the large cross section, (2) glossy wings, and (3) the low frequency (many Odonata have fundamental frequencies in the 50–100 Hz ranges). The hour of observation was late; however, temperature during this field campaign was exceedingly hot causing minimal insect activity at noon. We have general indications of Odonata activity in the evening. In the polarimetric cases, we have estimated the diffuse contribution by a linear gain on the depolarized band according to the DoLP.

### 2.6 Laser profiling

An industrial laser profiler model Gocator2040 from LMI3D was installed on the same translation platform as the hyperspectral cameras. The device emits a laser sheet at 630 nm perpendicular to the insect board. The instrument determines range by triangulation and returns a point cloud.

## 3 RESULTS: COMPARISON OF ODONATA WIPS AND VISION IN OPTICAL DOMAINS

### 3.1 Prospects of perceiving WIPs in the spectral domain

To test whether the thickness of the wing yields a specular reflection that can be perceived by the narrow red bands found in some Odonata species, we simulated fringes from thin film theory. The specular reflection is a coherent scattering process. In other words, the scattering patterns are governed by the memory of the impinging light's directionality of propagation, its polarization, and its phase. As the phase is preserved from the coherent specular reflection from both the first air–chitin interface and the second chitin–air interface, the resulting superimposed waves may either interfere constructively or destructively as a function of wavelength (Stavenga, 2014; Yin et al., 2006), membrane thickness, angle of incidence, and observation in respect to the surface normal (these angles are identical for specular reflections). The result of the thin film phenomena is that the specular reflection is characterized by spectral fringes across the spectrum of light (Stavenga, 2014; Yin et al., 2006). The finesse of the fringes can be exceptionally sharp for optical devices with high reflectance such as the Fabry–Pérot interferometer (James, 2007), but for insect wing reflectance is low and waveform is close to sinusoidal (Stavenga, 2014). The periodicity is constant in the frequency domain, so fringes are chirped reciprocally in wavelength domain. This implies that spectral fringes are sharp in violet and dull in red (Stavenga, 2014). A sharp fringe from a thick membrane is thus most convenient to spectrally resolve by a long‐wave narrow band. From our *Literature Review*, we present an overview of center wavelength and width of the longest band of a number of species in Figure 1a (Briscoe & Bernard, 2005; Briscoe & Chittka, 2001; Bybee et al., 2012; Eguchi, Watanabe, Hariyama, & Yamamoto, 1982; Futahashi et al., 2015; Kelber et al., 2003; Stavenga, 2002). From *Thin Film Numerical Calculations* (Stavenga, 2014; Yin et al., 2006), we added colored lines to indicate the corresponding fringe width from given chitin membranes at 56° Brewster angle (Stavenga, 2009). Whereas humans resolve thin film effects from approximately a half a micron thick membranes (See Figure 1a), we expect Odonata bands to resolve fringes from membranes up to ~2.4 μm thickness based on the width of their red bands. The same thickness that can be resolved by the narrow red bands found in some odonates is reported for Odonata wings in literature (Stavenga, 2014) and is also found in our data (see below). The ability to resolve and extract a spectral feature from the visual environment by a FWHM value should not be confused with the ability to discriminate wavelengths of monochromatic light (Koshitaka, Kinoshita, Vorobyev, & Arikawa, 2008). The latter is given by a much smaller Δλ value relating both to the band overlap and steepness and to the intensity resolution. That value can be measured in laboratory experiments, but is irrelevant in natural environments.

**Figure 2:**
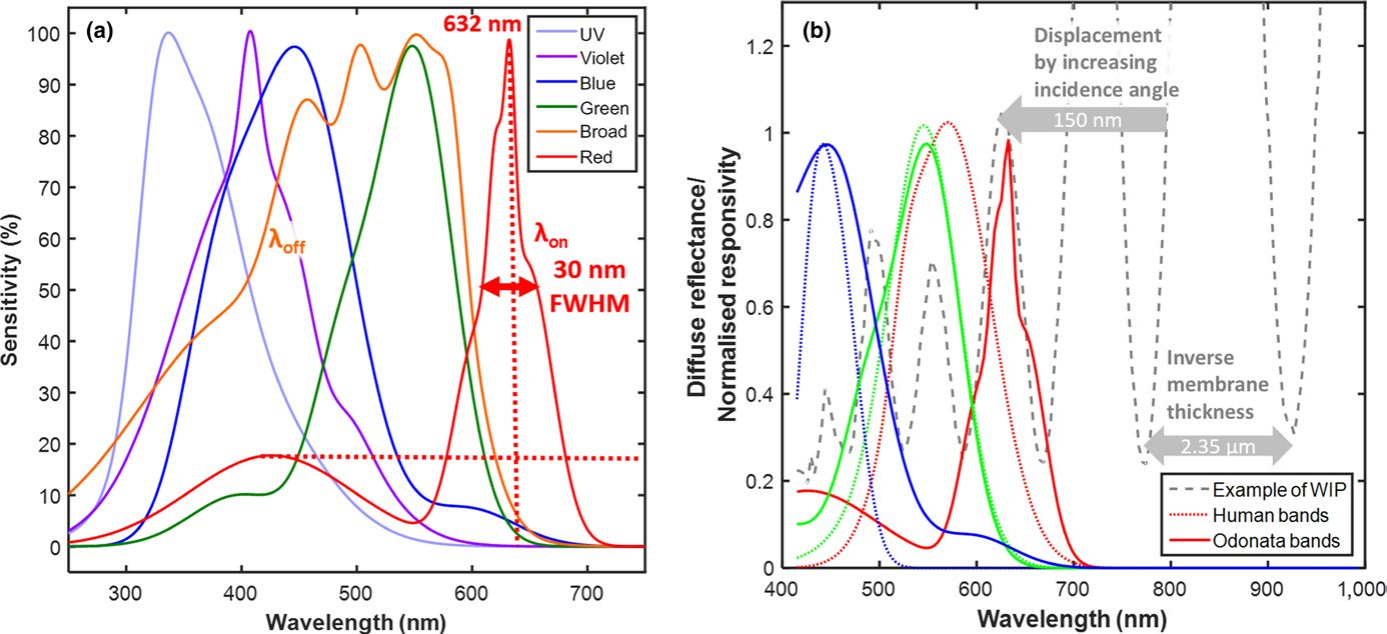
(a) Spectral bands of *Hemicordulia tau*, adopted from literature (Yang & Osorio, 1991). Bands are reproduced by a multi‐Gaussian fit to the original graphical material. The bands are not orthorgonal (they overlap), and the red band has a shoulder in the blue region, and the shoulder could be linearly unmixed through neural inhibition by the blue and violet bands. The broad band, λ_off_, and narrow band, λ_on_, index the fringes differentially. (b) Example of a spectral fringe measured from an Odonata wing. It is demonstrated how the human red band integrates over two fringes, whereas the narrow long‐waved band of some Odonata accurately samples one fringe of the thick wing membrane. As a result, humans do not perceive thick film effects, but certain Odonata may. The fringe is recorded at 22° incidence, the near‐infrared region is included in the plot because structural spectral features in this region would displace into the red region with increasing incidence angles. Examples where the spectral fringe dips at the red band peak can also be encountered, but both cases depend on the incidence angle and can displace some 150 nm from 0° to 70°, much beyond the bandwidth of 30 nm

It is well known that the natural variance of brightness (geometrical details) is much larger than the variance of chromaticity (commonly chemical composition). Brightness and chromaticity are often neurologically wired in different channels (Warrant & Nilsson, 2006). For a spectral signature to differ in the chromatic channel, it must contribute differentially to the spectral bands. Odonata have multispectral vision with up to six spectral bands (Futahashi et al., 2015; Huang, Chiou, Marshall, & Reinhard, 2014), and a differential contribution between any of these bands would imply a distinct chromaticity. The red spectral band of *S. rudicundulum* (Meinertzhagen et al., 1983) and *H. tau* (Yang & Osorio, 1991) are centered at particularly long wavelength (Bybee et al., 2012) ranging up to 632 nm and are the narrowest known bands (see Figure 2a). The narrow width is proposed to arise from neural inhibition (Yang & Osorio, 1991), but there are other potential mechanisms in insect vision. For instance, Tabanidae (Diptera) have been shown to narrow their bands by spectral filtering (Douglas & Marshall, 1999) including interference filters (Miller, 1979) which theoretically could provide much narrower bands (because the nature is structural rather than molecular). Therefore, in this report, the term “*band”* is used instead of “*receptor*,” because this is to emphasize the effective wavelength region, that is, the throughput of the whole visual system rather than the molecular rhodopsin absorption on the cellular and neurological component. In technology such as differential absorption lidar (DIAL; Mei & Brydegaard, 2015), sharp spectral features are assessed in the environment by a λ_on_ and a λ_off_ band, where one band is sensitive to an absorption feature and the other band is insensitive. Similarly, one way to differentially index spectral fringes spreading across the spectrum is to sample them with one broad spectral band and one narrow band, and the narrow red band may provide such a possibility in Odonata (see Figure 2a).

In Figure 2b, we present an example of a WIP spectrum from *Hyperspectral Imaging* indicating how a fringe from a 2.35‐μm‐thick Odonata wing produces a signal in the region of the red narrow band from the *Literature Review*. For comparison, human bands are included to illustrate why these fringes cannot be resolved by humans. We conclude that the Odonata with narrow red bands have the potential to differentially index fringes from specular reflections from the wings of conspecifics in the spectral domain.

### 3.2 Prospects of perceiving WIPs in the spatial domain

**Figure 3:**
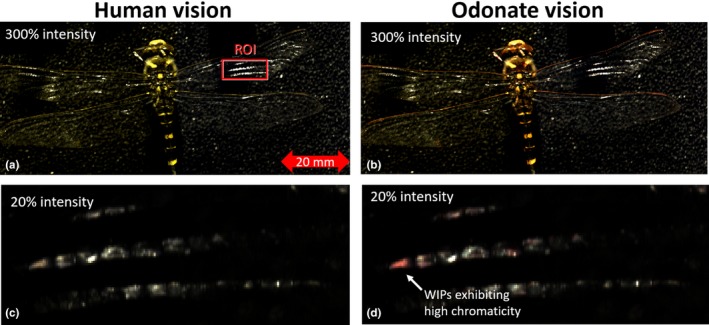
Hyperspectral images of a male *Cordulegaster boltonii*. (a) Projected on the human spectral bands, (b) projected on the three Odonata bands with the highest center wave length from Figure 2b. When comparing close‐up images (c and d), several reddish cells appear as the red bands narrows down. Because of the intense magnitude of specular reflections, the lower images are attenuated

**Figure 4:**
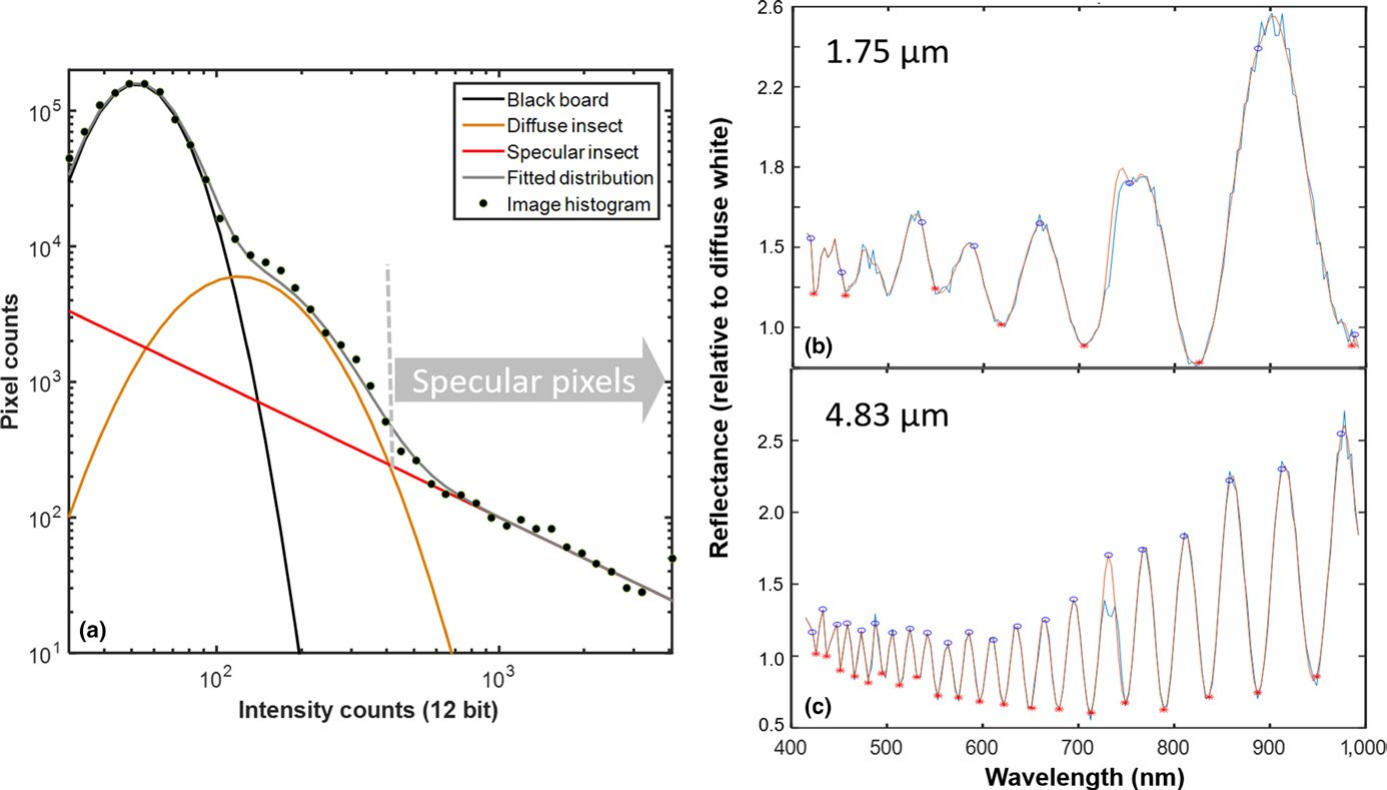
Selecting specular pixels and fringe fitting. (a) Image histogram of dragonfly on black background from Figure 3, the brightest 1,213 pixels of high magnitude were analyzed spectrally, and 95% of these pixels displayed spectral fringes of wing interference patterns. (b and c) Examples of thin and thick membrane segments in the image, respectively. Peaks and dips were identified in order to determine the free spectral range and membrane thickness in every specular pixel

**Figure 5:**
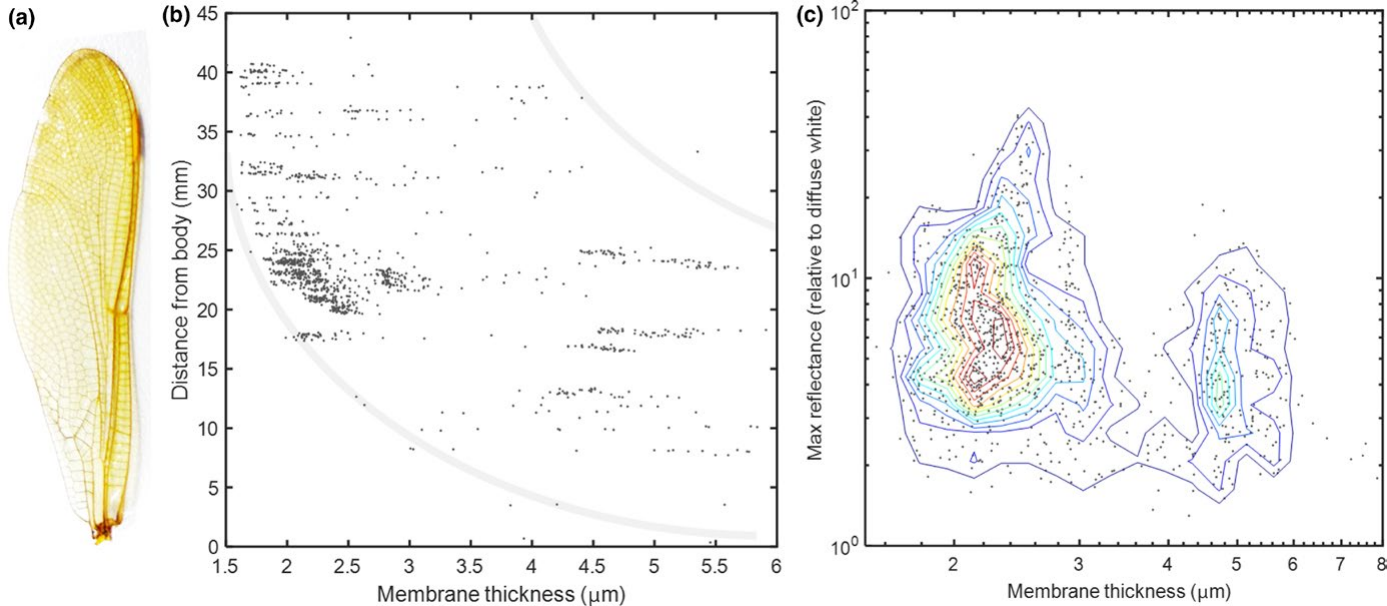
(a) Analyzed male *Cordulegaster boltonii* wing (b). Membrane thickness as function of distance to thorax (same scale as 5a). (c) Scatterplot of membrane thickness and reflectance magnitude. Specular reflections exceed 4,000% compared to diffuse white. The main statistical mode of membrane thicknesses appear at 2.34 μm. This value closely matches the estimated resolvable thicknesses in Figure 1a

**Figure 6:**
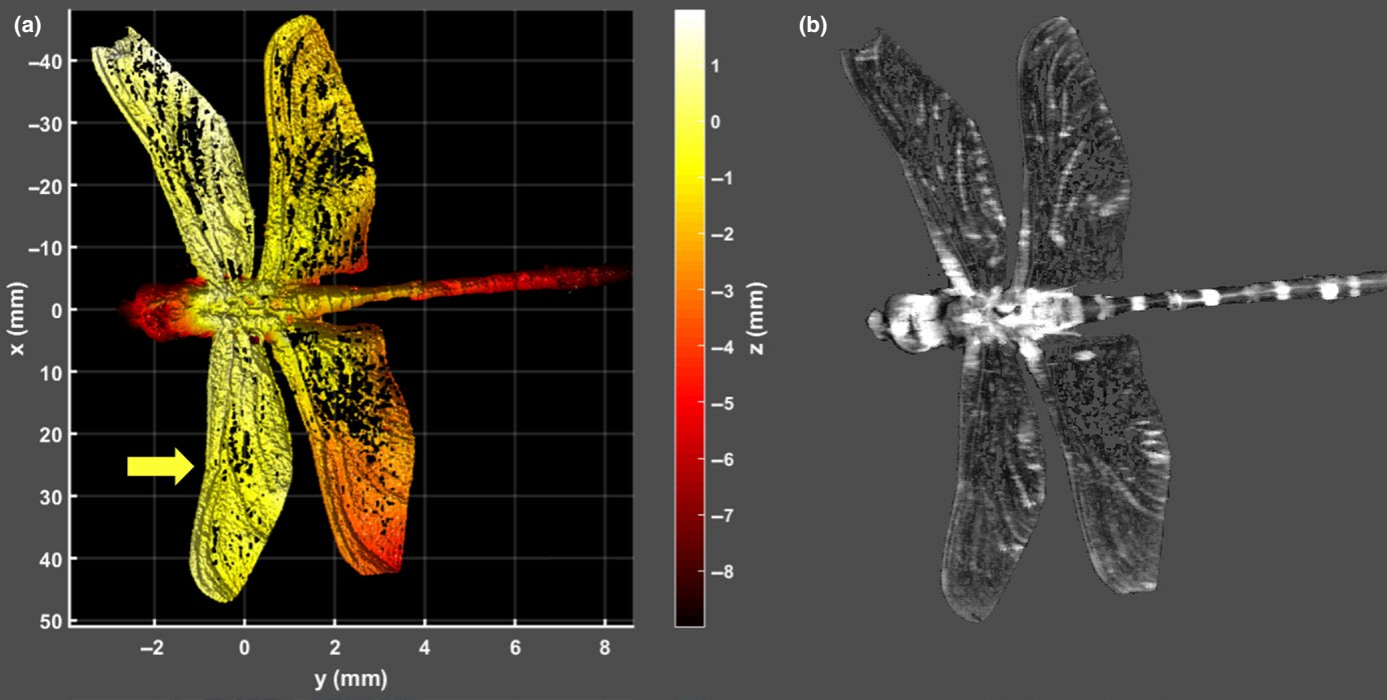
(a) Laser profiling shows wing flatness of a male *Cordulegaster boltonii*; thus, the majority of the wing would produce a specular reflection at a specific angle. The 3D point cloud data are presented by color coding the depth along the optical axis. Point cloud coordinates are relative to the center of the thorax. (b) Brightness image at 630 nm from the same instrument coinciding with the narrow red spectral band found in some Odonata

A second line of inquiry to address whether Odonata with narrow red vision bands can obtain information from specular wing reflections is the spatial resolution of their vision. The compound eyes of Odonata vision provide a high angular resolution (down to 0.2° in dorsal region) compared to other insects (Land, 1997), but low compared to humans (>0.02°). This implies Odonata with narrow red bands can resolve reflections from individual wing segments down to 200 μm while in tandem at 5 cm separation, whereas a specular reflection from a whole wing can be resolved at distances up to 12 m. These estimates of spatial resolution are based on values from *Anax junius* (Land, 1997), whereas acuity for other Odonata such as *Sympetrum* are 2–3 times poorer (Land, 1997). The resolution is highest in dorsal direction (Labhart & Nilsson, 1995) which is close to zenith for Odonata in tandem. To investigate whether the spatial variation in Odonata wing thickness can be perceived by their visual systems, we performed *Hyperspectral Imaging* (Figure 3). The specular pixels were selected by a threshold in the image brightness histogram (Figure 4a). A fringe was fitted to each specular pixel, and corresponding membrane thickness was derived (see Figure 4b,c). Odonata wings are thinner toward the tips (Combes & Daniel, 2003), and each individual segment in the wing membrane can have a specific thickness. The spatial distribution of membrane thickness can be observed by human color vision in dark field microscopy on insect species with thinner wings. These patterns are referred to as wing interference patterns (WIPs; Shevtsova et al., 2011). We find that wing membrane thicknesses presented vary from 1.5 to 6 μm. The most frequently observed thickness was 2.3 μm (see Figure 5), similar to effective thicknesses previously reported for Odonata (Stavenga, Leertouwer, & Wilts, 2013). In Figure 6, *Laser profiling* of the same left forewing display less than 1 mm deviation from a flat plane across the entire surface is shown.

In conclusion, we find that the spatial resolution of Odonata is such that the species with narrow red bands potentially can resolve individual wing segments while in tandem, and that specular reflections from whole wings of conspecifics flying at several meters distance.

### 3.3 Prospects of perceiving WIPs in the intensity domain

As a third line of inquiry, we address whether WIPs are likely to be a major contribution to the visual impressions of Odonata with narrow red bands. Compound eyes constantly stare directly into the sun, and this is bound to be the brightest perceived contribution. The magnitude of the reflected light from WIPs is governed by the Fresnel equations which are polarization‐dependent. As refractive index for chitin is higher than for water (Stavenga et al., 2013) and because the membrane has two interfaces, this implies that the intensity of specular reflection from wings is some five times greater than from water bodies. However, some Odonata wings have been demonstrated to have gradient refractive index rather than a step function, and this feature diminishes the reflectance (Stavenga, 2014). From *Hyperspectral Imaging* measurement presented in Figures 4a and 5c, we observe WIPs reaching magnitudes of 40 times Lambertian diffuse white reflectance. These magnitudes are obtained at a low 22° incidence and will be even larger at Brewster's angle. Change Following the magnitude of specular reflection from water bodies, the diffuse reflectance from vegetation and water typically constitute low reflectance of 10% and 5%. The magnitudes are listed in Figure 1b, but obviously these magnitudes are coarse estimates and subject to large variance. Hence, the overall contribution of the specular reflections to the visual impressions of the Odonata with narrow red bands is likely to be major, only second to the solar disk.

### 3.4 Prospects of perceiving WIPs in the polarization and angular domains

**Figure 7:**
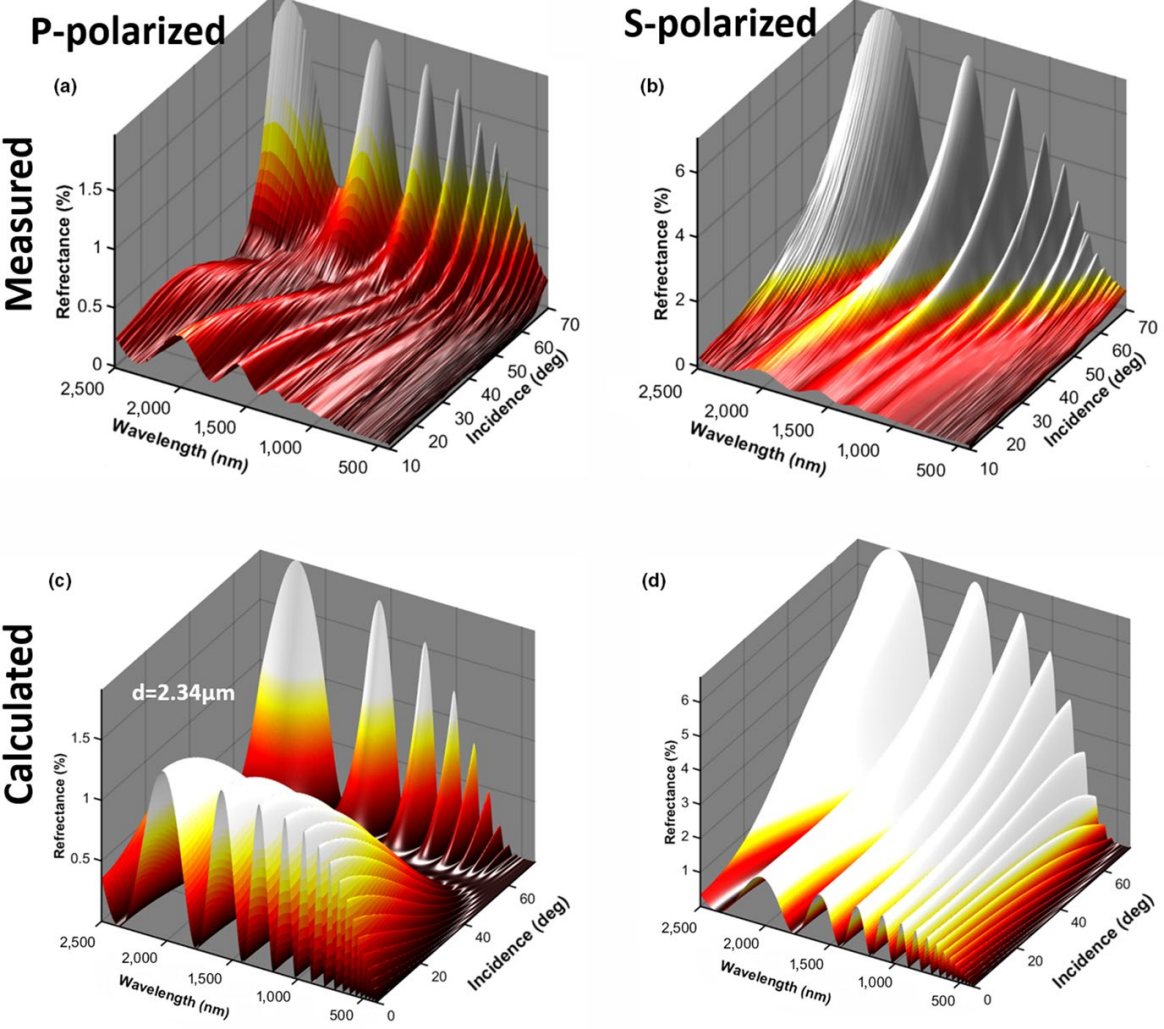
(a and b) Measured ellipsometry for a male *Cordulegaster boltonii* compared to a simple thin film model (c and d). The main discrepancy can be explained by a gradual refractive index change over a couple of hundred nanometers (Stavenga, 2014). The effect of this gradient attenuates modulations toward shorter waves and also displaces peaks. The best fit was encountered at 2.34 μm membrane thickness, which closely matches the estimated wing thickness values in Figures 1a and 5b from literature (Stavenga, 2014) and push broom imaging, respectively

Fourth, we examined whether Odonata with narrow red bands are likely to be able to observe WIPs in the polarization domain. While the direct sunlight is entirely unpolarized, specular reflections can pertain to a large degree of linear polarization. If the light impinges at Brewster angle (57° for chitin around 600 nm; Leertouwer et al., 2011), the resulting WIP is close to linear polarized. A polarization vision allows Odonata to detect coherent scattering at large incidence angles also known as specular reflections, for example, from water body surfaces and wing membranes. We performed spectral goniometric *Ellipsometry* on wings of a male *C. boltonii* to investigate their polarization properties (see Figure 7). The reflectance can roughly be described by *Thin Film Calculations* (Yin et al., 2006) and the Fresnel equations. The main source of discrepancy from this simple model is the gradient refractive index (Stavenga, 2014). From Figure 7, we understand that the Odonata with narrow red bands experience highly linearly polarized spectrally fringed light when observing specular reflections close to Brewster's angle, even from whole wings observed from a distance.

**Figure 8:**
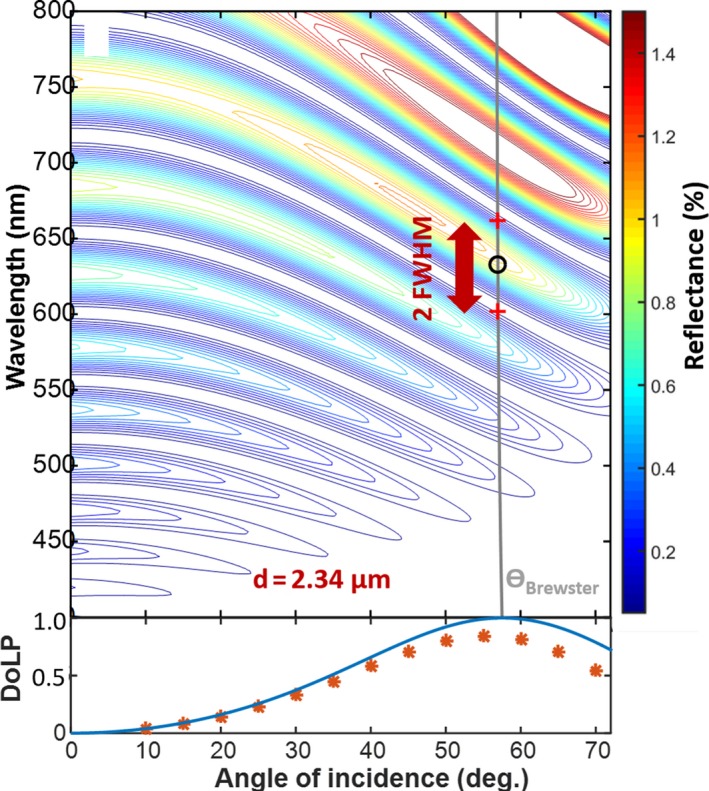
(a) Contour plot of the fitted model for S‐polarized reflectance. At Brewster angle, where the degree of polarization peaks, the band center and width are indicated. (b) The degree of linear polarization from Fresnel equations and measured values from ellipsometry. Experimental values reach 85% at Brewster angle

Interestingly, the narrow far red receptors of *H. tau* are encountered in the frontal part of the ventral eye (Yang & Osorio, 1991). In the study of *S. rubicundulum*, it is noted that the far red band is the only receptor on the ventral side displaying significant polarization sensitivity (Labhart & Nilsson, 1995), a feature common to the UV and blue receptors on the dorsal eye. The males have deep red pigmentation, and the red band is proposed to serve the purpose of contrasting conspecifics against natural background in *H. tau* (Yang & Osorio, 1991), but this hypothesis does not explain red narrow band polarization discrimination. Apart from *H. tau*, there are other cases of insect species with sexual dimorphism, where females have additional long‐wave receptors (Bernard & Remington, 1991). This was interpreted as adaptive for assessing wing coloration. From *Ellipsometry,* we conclude that WIPs reach a very high degree of linear polarization (DoLP; Figure 8). From *Literature,* we conclude that Odonata red bands pertain to polarization discrimination, and the red band receptors are located on the frontal side of the eye. These facts are consistent with Odonata with narrow red bands being able to perceive WIPs in the polarization domain.

### 3.5 Prospects of perceiving WIPs in the temporal domain

**Figure 9:**
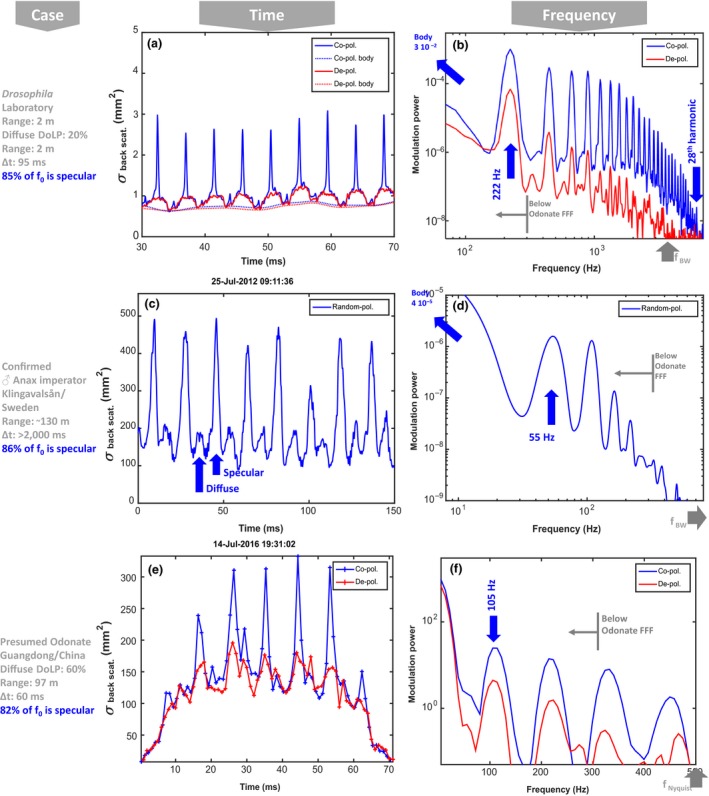
Polarimetrically resolved in vivo optical modulation signature of insects with glossy wings. Blue curves show co‐polarized light including specular and diffuse contributions. The red graphs show diffuse contributions estimated from depolarized light and a linear gain (inverse DoLP values). Δt denotes probe volume transit time, and *r* denoted detection range. The backscatter cross section values correspond to white diffuse Lambertian target. (a and b) Highly resolved *Drosophila melanogaster* laboratory recordings, specular reflections appear as rapid spikes in the temporal domain. (b) In the frequency domain, we observe up to 28th discernable harmonic at 6.2 kHz from the coherent part (limited by instrument −3 dB bandwidth which is indicated). (c and d) modulations from a male Anax Imperator over a stream in Sweden. (e and f) Polarimetric lidar backscatter from a presumed Odonata over a rice pond in China. Note the comparable cross sections in (c, d) and (e, f). In all, fundamental frequency, f_0_, is within flicker fusion frequency of Odonata and more than 80% of the f_0_ arise from the specular component carrying fringed wing interference pattern signatures

As a fifth test, we addressed whether WIPs could be temporally resolved by the vision of Odonata with narrow red bands. As discussed above, these Odonata are likely to be able to spatially resolve wings of conspecifics up to a distance of ten meters. Odonata wings can generate a specular reflection from the entire surface, see Figure 6a from *Laser Profiling*, and the WIP prevails when spatially integrating the whole wing, see Figure 7a,b from *Ellipsometry*. Odonata can alternate between both gliding (Wakeling & Ellington, 1997b) and flapping flight behavior (Wakeling & Ellington, 1997a). In terms of transmitting WIPs to a distant conspecific the flapping flight is the most challenging to capture. The specular reflections from free‐flying insects with glossy wings appear as a brief flash (Schultz & Fincke, 2009) in the temporal domain, see Figure 9a,c,e from In Situ *Modulation Spectroscopy*. These rapid flashes or spikes in the time domain constitute a high number of harmonic overtones (up to 28 discernable in Figure 9b) in the frequency domain, see Figure 9b,d,f (harmonic overtones are simply an alternative description of rapid features on a repetitive waveform). The number of harmonic overtones is limited by instrument bandwidth, and the inherent number of harmonics is likely higher. In comparison with the specular contribution, which is co‐polarized, the depolarized diffuse contribution only displays eight discernible overtones. Figure 9a,b illustrates laboratory high‐resolution recordings (Brydegaard, 2015) of a fruitfly, *Drosophila melanogaster*, with a fundamental frequency of 215 Hz. Figure 9c,d displays the modulation signature from a male *Anax imperator*. The signal is retrieved by remote dark field spectroscopy (Brydegaard et al., 2016; Runemark et al., 2012) from a distance of 130 m over a stream in southern Sweden. The species and sex are confirmed from video recordings. The dark field method currently does not allow polarimetric investigation, but diffuse and specular wing contribution is estimated from the nonspiky parts of the waveform. The fundamental frequency is at 55 Hz, and several of the harmonics are within Odonata flicker fusion frequency (FFF). In Figure 9e,f, a presumed Odonata is presented from polarimetric lidar data (Zhu et al., 2017). This observation was at 97 m range over a rice pond in southern China. The observation hour is late for Odonata; however, we have general confirmation of Odonata activity at the time, and also, the area is subjected to artificial lighting. As can be seen, reflections from species with glossy wings produce similar high harmonic content, but rescaled by the fundamental wingbeat frequency. Odonata have lower fundamental frequencies in the range 50–100 Hz. Specular signals would thereby contribute to frequency content up to at least 3 kHz, and hence, modulation in the range of 1–3 kHz can be attributed mainly to specular reflections. Nevertheless, more than 80% of the fundamental tone can be attributed the specular component. The specular flash from Odonata wings can be observed with high speed cameras; however, as RGB cameras mimic the human vision, WIP flashes appear white on such recordings (BBC, 2013).

In comparison with the modulation frequency content described above, temporal bandwidth (FFF) of Odonata has been reported to exceed 200 Hz (Inger, Bennie, Davies, & Gaston, 2014; Ishizawa & Arai, 2003; Miall, 1978). There are some reports of neurological temporal post processing such as band pass filtering in Odonata (Ruck, 1961), as well as Reichardt detectors and similar motion mechanisms (Gonzalez‐Bellido, Peng, Yang, Georgopoulos, & Olberg, 2013). Hence, it is possible that Odonata vision in species with narrow red bands can resolve the fundamental frequency of flying conspecifics from a distance; however, their vision do not have the necessary temporal bandwidth to resolve the >1 kHz range where the tones are entirely specular. As the specular reflection is at least one to two orders of magnitude more intense than the diffuse part, even a temporally smeared spike on the wingbeat waveform, with kHz content, would make a significant contribution to the fundamental frequency perceivable within the bandwidth of Odonata vision. In Figure 9, we demonstrate that more than 80% of the fundamental tone is contributed from the specular component (WIPs), and this is proved by using polarization or waveform features. Reports on the temporal bandwidth in Odonata vision are somewhat old but indicate flicker fusion frequencies (FFF) in the range 200–300 Hz (Ruck, 1961). Such bandwidths were further confirmed through personal communication with Dan‐Erik Nilsson and Eric Warrant.

When considering the degree of linear polarization and spectral fringes from whole wings of this same contribution, we conclude that it is possible to resolve the fundamental wingbeat frequency for Odonata with narrow red bands and that WIPs from other Odonata flying at a distance make a dominant contribution to the fundamental tone that Odonata can see, despite that the temporal details (overtones) are not resolved. This is in accordance with available literature (Schultz & Fincke, 2009).

## 4 CONCLUSION AND PERSPECTIVES

We conclude that wing interference patterns (WIPs) from thick film interference may, judged by the extensive experimental data examined, be spectrally revolved by the vision of Odonata. Furthermore, the magnitude of the WIP signal is considerable compared to surroundings, the degree of linear polarization is greater than that from the surroundings, and polarization can be sensed by Odonata with narrow red bands. Interestingly, Odonata may be able to resolve specular reflections from single wing membrane segments when in tandem and from whole wings at distances up to 10 m. Both Odonata vision literature (Briscoe & Chittka, 2001; Bybee et al., 2012; Futahashi et al., 2015; Kelber et al., 2003) and *Thin Film Calculations* suggest an effective wing membrane thickness of 2.3 μm would produce WIPS that can be perceived by the Odonata visual system for Odonata with narrow red bands, and this is also the wing thickness we measure in this study with *Hyperspectral Imaging* and *Ellipsometry*.

*Cordulegaster boltonii* and *A. imperator* which are analyzed in this study are not among the species whose narrow red bands are described in literature, *S. rudicundulum* and *H. tau*. There is, however, no evidence that *C. boltonii* or *A. imperator* would not have narrow red bands, and there are few, if any, reasons to assume that the coarse wings of *S. rudicundulum* and *H. tau* would not exhibit equally sharp spectral fringes as the ones described here.

The fact that narrow red band receptors are sensitive to polarization and are located in the female frontal part of the ventral eye is consistent with the hypothesis that the spectral width of receptors is advantageous to evaluate wing membranes of males when in tandem position. We have also discussed the prospects of sensing WIPs at a distance from flying conspecifics. Although WIPs may not be resolved temporally, we estimate that WIPs still make a significant contribution to the fundamental frequency which is resolved by Odonata vision for species with narrow red bands.

In addition to the independent lines of evidence from various domains suggesting that Odonata could be able to perceive WIPs from wings of conspecifics, tests are needed to establish the behavioral and adaptive significance of the narrow red bands. Systematic searches for the presence of the narrow red bands in Odonata and behavioral studies in the taxa that have these bands would be needed to evaluate potential adaptive value. Below, we list situations in which perceiving WIPs potentially could be beneficial.

There would be several potential advantages if the narrow red bands of Odonata would allow for perceiving wing properties of conspecifics. First, if specular conditions could provide additional species and lineage information of use in identification of competitors and/or potential mates (Katayama, Abbott, Kjærandsen, Takahashi, & Svensson, 2014), it allows earlier response to intrusion on the territory or unwanted courtship. Second, poorer condition, flight skills, and frequent collisions are reflected as wear and flaws on the wings (Combes, Crall, & Mukherjee, 2010) and specular conditions are ideal to evaluate flaws and surface roughness. Specular testing is known from quality testing of optical elements and could analogously be exploited by females to evaluate flight performance. Third, refractive index increases the glossiness of dragonfly wings and alters the free spectral range of the fringes. In some Odonata species, newly emerged and immature individuals have glossy wings (Brydegaard, 2015), and there are indications that age is related to refractive index for, for example, *Anopheles gambiae* (Mayagaya et al., 2009; Peiris, Drolet, Cohnstaedt, & Dowell, 2014). As the compound eyes of Odonata look directly into the sun simultaneously as they would see a specular reflection, information on the angular difference is available to them and therefore also the Brewster angle and the refractive index. Potentially, this could enable Odonata to evaluate age of competitors and potential mates. In terms of geometrical arrangement in a detection scenario, the chromaticity of WIPs from distant conspecifics solely depends on the observed angular difference between the sun and the conspecific, φ (See Figure 1b). As the angular resolution of the Odonata vision is roughly the same as the aperture from the sun disk, the chromaticity from consecutive flashes from a distant conspecific is preserved (no iridescence). Another potential advantage to WIPs that hypothetically can only be perceived by species with narrow red bands would be that signaling between conspecifics could be achieved without an increased risk of predation from predators with different vision systems. Then, it would be a “private channel” (Cummings, Rosenthal, & Ryan, 2003), comparable to, for example, pheromones (O'Connell, Beauchamp, & Grant, 1986). Although it is generally considered that Odonata wing membranes are too thick to produce fringes visible to the human eye, there are numerous studies showing that thin film effects serve the purpose of sexual selection in birds (Hill & McGraw, 2006b), and WIPs are thought to play a role in sexual selection of some insects (Katayama et al., 2014; Schultz & Fincke, 2009). Testing whether mates with wings of the right thickness are preferred over, for example, mates with experimentally thinned or thickened wings or altered polarizing properties would shed light on whether WIPs are used in mate choice in Odonata, as is shown in *D. melanogaster* (Katayama et al., 2014). Color manipulation protocols of wings have yielded interesting insights in *Calopteryx* mate preferences (Svensson et al., 2010), and experimental alteration of wings is hence feasible.

In conclusion, we have provided several lines of evidence suggesting that investigating whether the narrow red bands of some Odonata species may be an adaptive character that enables them to use spectral information from thick film inference is a very promising future line of research. If WIPs are indeed exploited by animals for recognition and mediated over distance in the visual environment, it may pose a significant technological advancement for the purpose of target classification in entomological surveillance (Brydegaard et al., 2016; Runemark et al., 2012) and entomological lidar (Brydegaard et al., 2014, 2017; Zhu et al., 2017) with direct value for biodiversity monitoring purposes.

## CONFLICT OF INTEREST

None declared.

## AUTHOR CONTRIBUTIONS

MB carried out multispectral imaging and laser profiling, noted the resemblance between WIPs and vision properties, produced graphical items, and drafted the manuscript. SJ and MB carried out modulation spectroscopy. MS carried out ellipsometry. AR contributed with expertise on odonate evolutionary ecology and partially wrote the manuscript. All authors revised the manuscript.
